# Histogenetics in Teaching the Complexity of Developmental Biology to Dental Students: A Study Merging Traditional and Current Approaches

**DOI:** 10.3390/dj14030177

**Published:** 2026-03-17

**Authors:** Camilla Sofia Miranda Kristoffersen, Camilla Elise Øxnevad Ziesler, Noora Helene Thune, Anna Tostrup Kristensen, Tor Paaske Utheim, Hugo Lewi Hammer, Amer Sehic, Alan Henry Brook, Qalbi Khan

**Affiliations:** 1 Institute of Oral Biology, Faculty of Dentistry, University of Oslo, 0316 Oslo, Norwayamer.sehic@odont.uio.no (A.S.); 2 Department of Medical Biochemistry, Oslo University Hospital, 0316 Oslo, Norway; 3 Department of Plastic and Reconstructive Surgery, Oslo University Hospital, 0316 Oslo, Norway; 4 Unit of Oral and Maxillofacial Surgery, Department of Otorhinolaryngology, Oslo University Hospital, 0316 Oslo, Norway; 5 Department of Computer Science, Faculty of Technology, Art and Design, Oslo Metropolitan University, 0316 Oslo, Norway; 6 School of Dentistry, The University of Adelaide, Adelaide, SA 5000, Australia; alan.brook@adelaide.edu.au; 7 Faculty of Social and Health Sciences, Inland Norway University of Applied Science, 2418 Elverum, Norway

**Keywords:** teaching genetics, digital learning, digital video-based tools, tooth morphology, dental anatomy

## Abstract

**Background:** Dental students need to qualify with a clear understanding of the continuum of biological development from the molecular (genetic, epigenetic and environmental interactions) to the cellular (morphogenesis and differentiation) to the emergence of the mature tissue or organ. Histogenetics provides a core component for this understanding. The aim of this study is to investigate whether a merged approach, combining traditional and recent methods, can enhance the teaching of histogenetics to dental students. **Methods:** This study blended traditional (lectures, drawings, microscopy) and recent approaches (flipped classroom elements, virtual microscopy, group-based poster construction, and interactive quiz-based discussion) to enhance student engagement and perceived learning in oral histogenetics. The intervention was delivered to master-level dental students across six core oral histogenetics topics. Teaching followed a structured three-phase model: Prepare (digital lectures and short microscopy-introduction videos); Engage (microscopy session and group-based poster creation); and Test and Discuss (teacher-led quizzing and discussion). Student perceptions were evaluated through an electronically distributed 17-item questionnaire at the end of the course. Items were grouped into self-evaluation, resources, and teaching method domains and rated on a five-point Likert scale. **Results:** A total of 45 of 51 students responded (88%). Across all domains, positive perceptions (Agree/Strongly Agree) predominated (*p* < 0.001). Self-evaluation items showed strong agreement for attendance and group contribution, with more variability in preparation time and motivation. Resources were rated highly, although the accessibility of physical guidance showed more mixed responses. The merged teaching method received strong endorsement, with students reporting engagement, enjoyment, ease of understanding, and clear emphasis on clinical relevance. **Conclusions:** The merged approach was perceived as pedagogically valuable and clinically meaningful by the students and appears to enhance perceived engagement, clarity, and relevance in oral histogenetics teaching. These findings support the adoption of blended, student-active methodologies to strengthen comprehension and promote clinically meaningful learning in oral histology.

## 1. Introduction

Genetic, epigenetic and environmental factors are involved in the complex system of interactive networks that give rise to the development of the dentition [1,2]. Interactions between factors at the molecular level lead to the development of cells and tissues that interact to give rise to the emergence of calcified, mature teeth. Dental students need to qualify with a good understanding of this process.

**Figure 1:**
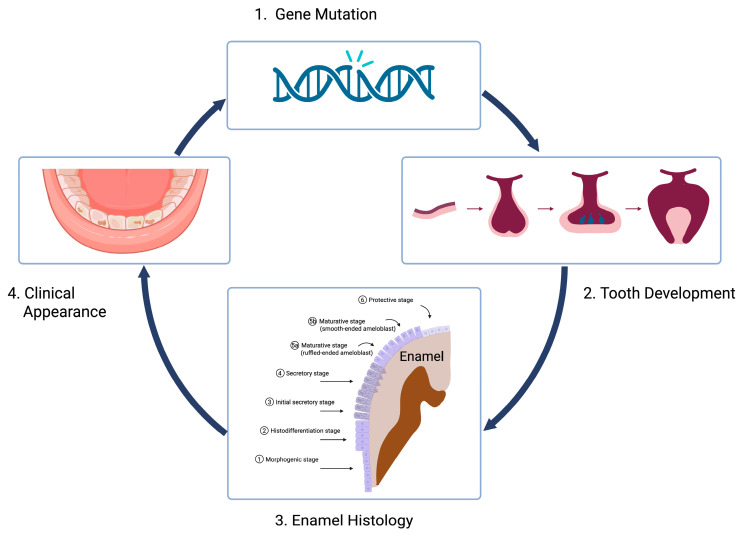
Linking structure, function and disease. Histology (3) bridges molecular genetics (1) and the origins and organization of tissues (2) to clinical significance (4) and is therefore critical for accurate diagnosis (4) and our understanding of disease (1).

It forms a basis for diagnosis and treating the variations and anomalies that are frequently seen in clinical practice. To enhance students’ understanding, histogenetics serves as a bridge between molecular genetics and gross anatomy. It facilitates an appreciation of cellular organ organization and the translation of molecular mechanisms into tissue and organ function [3,4]. Beyond its role in linking structure and function, histology carries substantial clinical significance. Its importance lies in the capacity to reveal the origins and organization of tissues, their differentiation into functional physiological structures, and their alterations under pathological conditions. As many diseases microscopically manifest before gross anatomical changes become apparent, histology provides a critical foundation for accurate diagnosis and for understanding disease progression [5] (Figure 1).

A solid command of histological principles is therefore indispensable for all current and future clinicians, as it enables the recognition of early lesions, the interpretation of diagnostic slides, and the integration of microscopic findings with underlying disease mechanisms [6].

Furthermore, the discipline of histology holds significant value in genetics, medicine and biomedical research. It provides a critical means of assessing tissue responses and evaluating biocompatibility, thereby ensuring both the safety and effectiveness of emerging therapeutic innovations [7,8]. Advances such as molecular genetic analysis, digital pathology, and high-resolution imaging rely on a profound understanding of histological principles, as they integrate microscopic structure with molecular and functional data, thereby enhancing diagnostic accuracy, research outcomes, and the development of targeted therapies [9].

Within odontological diagnostics, histology occupies a central role, as research has shown that clinical assessment alone often leads to diagnostic inaccuracies, thereby underscoring the necessity of histological examination in conjunction with clinical evaluation [10,11]. In practice, accurate diagnosis relies on a comprehensive approach that integrates clinical findings, radiographic imaging, and histological analysis. Knowledge of normal tissue architecture and histopathology is crucial for identifying pathological changes, distinguishing between differential diagnoses, and ensuring effective and precise treatment [12,13].

Histology is a visually oriented discipline that relies on microscopy and specialized staining techniques to identify and differentiate cell types and tissue architectures. Through the examination of stained sections at varying magnifications, students gain insight into cellular organization and its role in shaping tissues and organs. Such analyses not only elucidate the developmental origins of tissues but also demonstrate how these structures contribute to physiological function and how they undergo pathological transformation [14].

Histology teaching is commonly structured as a two-component system. The first component is the theoretical foundation, delivered through lectures, self-study, or flipped classrooms, focusing on memorization of cellular and tissue structures as well as links to embryology, molecular biology, and genetics. The second component is practical, involving active learning in laboratory sessions with light or virtual microscopy, where students develop recognition and interpretation skills [15,16].

Oral histology is a specialized subfield of histology concerned with the microscopic investigation of the structure, organization, and function of oral tissues and their associated components. Oral histology teaching can be delivered using a variety of methods, each uniquely contributing to students’ learning outcomes [16]:

- Didactic lectures, in which the instructor presents structured knowledge through, for example, PowerPoint presentations, blackboard instruction, or digital lectures.
- Light microscopy, where students use physical microscopes and glass slides to observe tissue structures and understand biological variation.
- Drawing microscopy images, where students draw what they observe under the microscope.
- Virtual microscopy, in which digitally scanned microscope slides are made available on computers or tablets.
- Group/team-based learning, where students work in small groups to discuss, solve problems, and reflect.
- E-learning elements, in which students use online resources such as e-books, websites, e-learning platforms, social media, podcasts, online tutorials, Massive Open Online Courses (MOOCs), and mobile applications.
- Quiz module and gamification, in which the lecture material is incorporated with quizzes, point systems, and game-like elements.
- Flipped classroom and online lectures, where students digitally engage with lecture materials prior to class, while class time is used for discussion, problem-solving, and application of knowledge.

Traditionally, histology education has relied on a combination of didactic and practical components, providing students with both a solid theoretical foundation and hands-on experience with tissue specimens [15]. However, these traditional methods can often encourage passive learning and offer limited opportunities for the development of critical thinking skills. Incorporating modern strategies, such as flipped classrooms, team-based learning, and digital tools, promotes active engagement, collaboration, and deeper cognitive processing. Hence, we recently proposed a blended model in which several methods are structured and chronologically combined and hypothesized that this dynamic teaching method may both engage and ignite interest in oral histology for the students [16].

The aim of this study was to implement the suggested blended teaching approach to oral histology for master’s students in dentistry at the University of Oslo (UiO) and assess this method through a student-perceptions-based questionnaire.

## 2. Materials and Methods

Teaching method: The entire student group was initially introduced to oral histology through traditional introduction lectures (regarded as “foundation-building themes”, such as embryology, genetics and developmental biology of the head). Then the students were introduced to the new learning format. The teaching was focused on six key topics:

1. Tooth development;
2. Dentinogenesis and Amelogenesis;
3. Microstructure of dentin, enamel and pulp tissue;
4. Root, cement and PDL (development and microstructure);
5. Tooth eruption and shedding;
6. Oral mucosa.

Each topic was allocated a specific session. The course was structured as a three-phase teaching model: Prepare, Engage, and Test and Discuss.

Prepare: One or two online lecture videos (between 2 and 3 h) were shared on the digital platform named Canvas roughly 4–6 days before the course day. On the day before the course, a shorter digital video (2–3 min long), developed by our department, was shared. This focused on introducing digital microscopy for the specific subject while utilizing phrases and knowledge introduced earlier in the online lectures.

Engage: At the beginning of the session, original sections were introduced and studied under the microscope together with teaching staff. This task was relatively brief, taking roughly 30 min in total. Students were then split into breakout groups of 4–5 and made a visual presentation of the sections, carefully designed to respect the developmental chronology. Students were supplied with colored pencils and markers, poster charts, colored papers, scissors, glue sticks, tape and other items to encourage and facilitate the creative display of the topic on a chart. They were given approximately 1 h to accomplish this task.

Test and discuss: After an hour, each group was visited by a member of teaching staff. Here, they presented their poster/flowchart, and an active quiz-based discussion was led by the teacher. Every student was included in testing their knowledge when quizzed about the drawings, actively leading to discussions where the whole group could fill in and participate. This session would last for about 10–15 min.

Student perception: To evaluate students’ perceptions of the teaching process and personal outcome, a questionnaire was developed and administered at the end of the course. The questionnaire was designed for self-reflection and evaluation of the resources and students’ experiences. This study was reviewed according to institutional guidelines and deemed exempt from formal ethical review, as it involved voluntary, anonymous survey participation. Completion of the questionnaire was interpreted as informed consent.

The online questionnaire was organized into three thematic sections:

1. Self-evaluation—This section had 4 items and prompted students to assess their own learning motivation and engagement.
2. Resources—The six questions in this section examined the adequacy, accessibility, and perceived effectiveness of the learning resources provided, including digital tools, course materials, and instructor support.
3. The teaching method (7 items)—This section focused on students’ perceptions of the newly implemented pedagogical approach.

All items were rated using a five-point Likert scale ranging from 1 (Strongly Disagree) to 5 (Strongly Agree). This study included master-level dental students enrolled in oral histology at the University of Oslo. Prior to entering the dental-specific track, students had completed foundational basic science courses together with general medical students during the first two years of the curriculum. The dental program then continues with three years of clinical dentistry training. Oral histology, together with oral genetics and developmental biology, is delivered after the separation from the medical curriculum and prior to the clinical phase.

The cohort was academically and sociodemographically relatively homogeneous. Most students were between 20 and 25 years of age, and sociodemographic variation is generally limited within the Norwegian educational context. However, approximately 80% of the cohort were female.

The questionnaire was electronically distributed to all enrolled students during the final week of the course. Participation was voluntary and anonymous, ensuring that students could express their opinions freely. A total of 45 of 51 students responded, making it a study based on 88% of the participants of the course.

Statistical analysis: Data were analyzed using descriptive statistics to present central tendencies and variability across responses. Mean scores and standard deviations were calculated for each section and individual item to identify patterns in student perceptions. To test whether students’ responses were generally above the Neutral category (i.e., leaning toward *Agree* or *Strongly Agree*), we used a one-sample Wilcoxon signed-rank test. This test is well suited for Likert-type data because it relies only on the ordering of the response options and does not assume that the steps between categories are equally spaced. A significance level of 0.05 was used in the interpretation of the statistical tests. All statistical analyses were done in the statistical software R version 4.5.0 [17]. Plots were made using the R package ggplot2 [18].

## 3. Results

**Figure 2:**
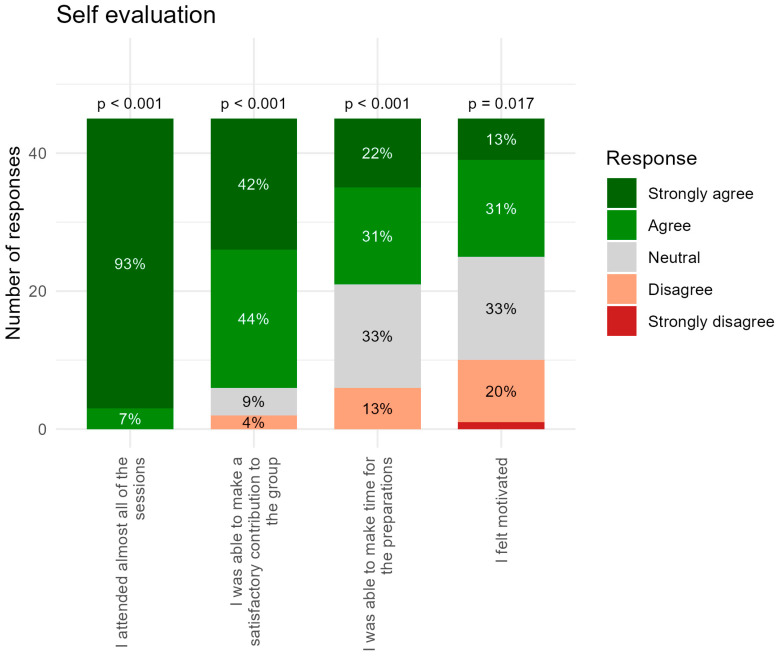
Student questionnaire response for self-evaluation section with four questions. The bars represent the questions with a five-level agreement scale (from Strongly Agree to Strongly Disagree) in which percentage response distribution is given as colored from green to red. *p*-values are calculated to test whether students’ responses were generally above the Neutral category.

Self-evaluation: Responses in the self-evaluation section indicated generally positive perceptions across all four assessed items (Figure 2). For “I attended almost all of the sessions”, responses were overwhelmingly positive, with 93% selecting *Strongly Agree* and the rest of 7% selecting *Agree*. The *p*-value indicates strong evidence that attendance was consistently high.

A similar strong statistical support was also present because people felt they contributed well in the item “I was able to make a satisfactory contribution to the group”. Slightly more variation than the attendance item was observed, with 42% selecting *Strongly Agree* and 44% *Agree*, with only 9% selecting *Neutral* and 4% *Disagree* (*p* < 0.001). This suggests that most participants felt confident about their contributions.

A more mixed response than for the first two items was observed for “I was able to make time for the preparations”. A substantial proportion (33%) reported Neutral, and 13% Disagree; however, a majority still agreed (22% Strongly Agree and 31% Agree (*p* < 0.001)). This indicates that preparation time was manageable for many but not all.

The most variable item was found in motivation. For “I felt motivated”, only 13% selected Strongly Agree and 31% Agree, compared with 33% Neutral, 20% Disagree, and a small 2% Strongly Disagree. The *p*-value (0.017) was higher than for the other items, suggesting more heterogeneous experiences, with some participants struggling to maintain motivation.

Overall, the students evaluated themselves positively across all four statements, with most responses falling into the Agree or Strongly Agree categories. Attendance and contribution were the strongest areas, whereas preparation time and, especially, motivation showed greater variability among participants.

**Figure 3:**
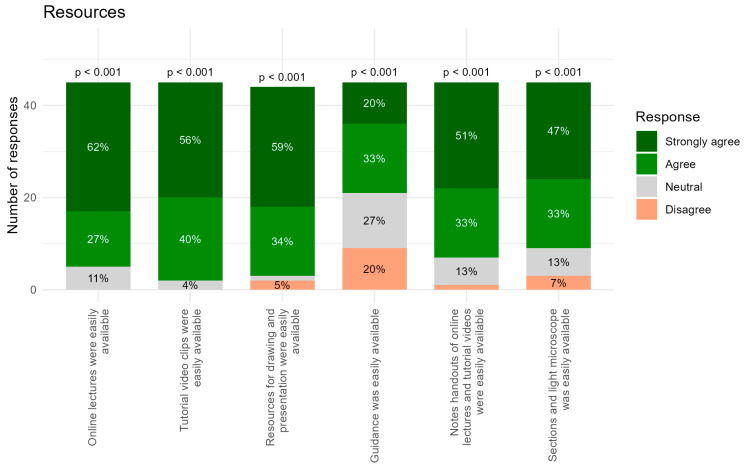
Student questionnaire response for the section of Recourses with six questions represented as bars with percentage distribution. *p*-values are calculated to test whether students’ responses were generally above the Neutral category.

Resources: For the statements “Online lectures were easily available” and “Tutorial video clips were easily available” (Figure 3), responses were overwhelmingly positive. A total of 62% and 56% selected Strongly Agree, respectively, with an additional 27% and 40% selecting Agree. Only small neutral proportions (11% and 4%) and no disagreement were reported. These results indicate that online learning materials were highly accessible.

Most participants also agreed that “Resources for drawing and presentation were easily available”. Here, 59% selected Strongly Agree and 34% Agree, with only 5% disagreeing and a small Neutral group (2%). This shows that the availability of tools and materials for drawing and presentation was perceived as very good, with only minimal variability.

The greatest variability in this section was observed for “Guidance was easily available”. Although a majority still reported positive experiences (20% Strongly Agree, 33% Agree), there were 27% neutral responses and 20% disagreement. Despite this spread, the overall trend was significantly positive (*p* < 0.001).

In line with the positive evaluations of online materials, “Notes/handouts of online lectures and tutorial videos were easily available” also received predominantly positive responses, with 51% Strongly Agree, 33% Agree, 13% Neutral, and 2% Disagree selections.

Participants likewise affirmed that “Sections and light microscopes were easily available”, with 47% selecting Strongly Agree and 33% selecting Agree. Only 13% were neutral and 7% disagreed, indicating generally high accessibility of physical study materials throughout the course.

Taken together, participants reported highly positive evaluations of the availability and accessibility of learning resources (Figure 3). Online resources were rated as easily accessible by nearly all respondents. Resources for drawing and presentation, as well as access to microscope sections, were also viewed very favorably, though with slightly more variation. Guidance availability showed the widest range of variability.

**Figure 4:**
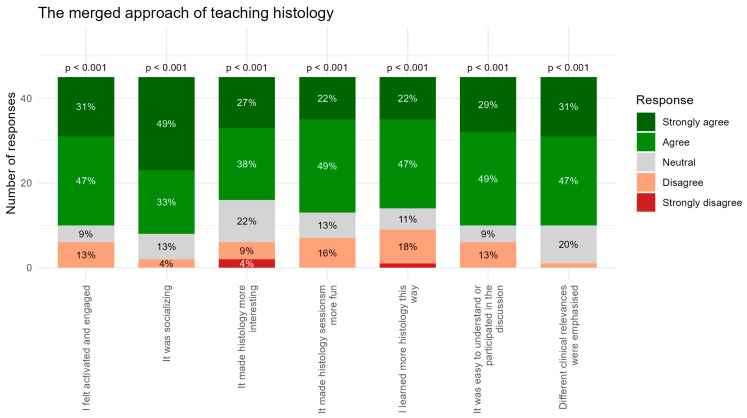
Student questionnaire response for the section of intervention method, with seven questions represented as bars. *p*-values are calculated to test whether students’ responses were generally above the Neutral category.

The teaching method: Participants reported high levels of activation and engagement during the merged histology sessions (Figure 4). For “I felt activated and engaged”, 31% selected Strongly Agree and 47% Agree, with only 9% selecting Neutral and 13% Disagree (*p* < 0.001). This indicates that the merged approach effectively stimulated participation for most respondents.

The merged approach was also perceived as socially supportive. For “It was socializing”, 49% selected Strongly Agree and 33% Agree, with a neutral proportion of 13% and a small amount of disagreement (4%). This suggests that most found the sessions socially engaging.

Interest in histology increased (compared to prior experience) for the majority of participants. For “It made histology more interesting”, responses showed 27% Strongly Agree and 38% Agree, with 22% Neutral, 9% Disagree, and 4% Strongly Disagree. The positive skew demonstrates a clear overall effect, despite a small proportion expressing reservations.

Participants agreed even more strongly that the merged approach made histology sessions more enjoyable. For “It made histology sessions more fun”, 22% Strongly Agree, 49% Agree, and 13% Neutral selections were observed, with 16% disagreeing, still resulting in a predominantly positive trend (*p* < 0.001).

Enhanced learning outcomes were also reported through “I learned more histology this way”. Here, 22% selected Strongly Agree and 47% Agree, with only 11% reporting Neutral and a combined 20% Disagree selection. While a minority disagreed, the overall perception strongly favored improved learning.

Ease of comprehension and participation in discussions was rated particularly highly. For “It was easy to understand or participate in the discussion”, 29% Strongly Agree, 49% Agree, and 9% Neutral selections were recorded, with 13% Disagree selections. The distribution shows a very strong positive consensus (*p* < 0.001).

Finally, participants overwhelmingly affirmed that the merged approach successfully made room for emphasizing clinical relevance. For “Different clinical relevances were emphasized”, 31% selected Strongly Agree and 47% Agree, with 20% selecting Neutral and very limited disagreement, indicating that clinical connections were clearly perceived and valued.

In summary, participants evaluated the merged approach to teaching histology highly positively across all items. They reported feeling activated and engaged, and many perceived the sessions as socially supportive. The approach was widely regarded as making histology more interesting and enjoyable and enhancing learning. Finally, participants strongly agreed that clinically relevant aspects were effectively highlighted.

## 4. Discussion

In this study the traditional dual-component model, didactic lectures combined with laboratory-based microscopy, was expanded to include more recent tools of learning, including flipped classroom elements, virtual microscopy, group-based learning with poster making, and interactive quiz and discussion modules. The clinical relevance was highlighted, underscoring the importance of histology in diagnostic reasoning and treatment planning.

**Figure 5:**
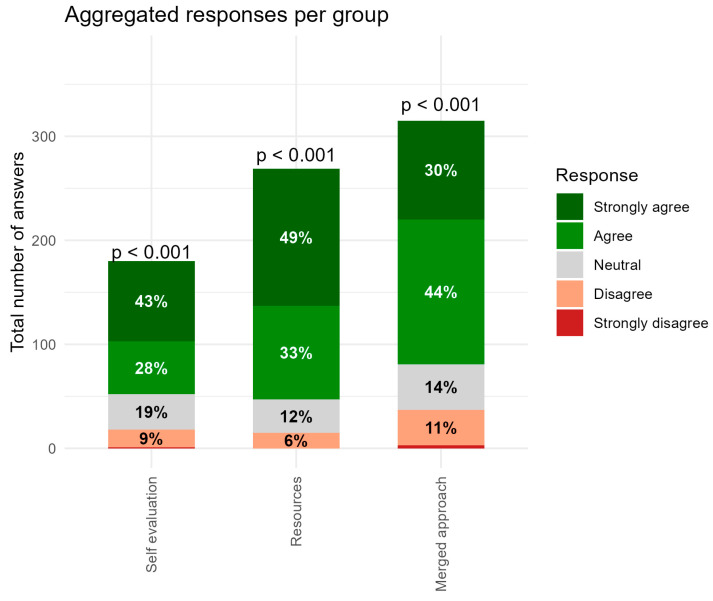
Aggregated responses per group, illustrating the distribution of responses across the three sections assessed: self-evaluation, resources, and the merged approach, in Figure 2, Figure 3 and Figure 4.

Figure 5 shows “Aggregated responses per group,” illustrating the distribution of responses across the three aspects assessed: self-evaluation, resources, and the merged approach. Across all groups, positive responses (Agree + Strongly Agree) constituted the majority (*p* < 0.001).

The self-evaluation group exhibits a predominantly positive pattern, with a combined 71% of respondents agreeing or strongly agreeing (Figure 5). Despite this overall endorsement, this group also displays the highest proportion of neutral and negative responses relative to the other two conditions, particularly regarding preparation time and motivation (Figure 2). One explanation for the lower ratings in preparation time may be related to the semester being densely scheduled, with substantial curricular demands from courses such as tooth morphology and macroanatomy, in addition to the proximity of major examination periods. Under such conditions, students may have found it challenging to allocate sufficient time for preparatory work, thereby negatively influencing their perceptions.

The lower motivation scores may reflect the nature of the learning activity itself. The presentation format required active participation, direct questioning, and ongoing engagement by the instructor, which can be demanding for students, particularly those who feel less confident or prefer more passive learning environments. We emphasize here that the profession of dentistry requires social skills, verbal skills, professional interaction and patient communication [19]. Activities that involve real-time quizzing and activate discussion may, for some students, heighten performance pressure [20] but should be understood as vital for their future professional life. Hence, even though the method may be pedagogically sound, it may result in reduced motivation for some learners. Consequently, the observed negative responses in motivation may stem less from the instructional or pedagogical quality and more from the demands inherent in highly interactive teaching formats. To address this issue in the future, there should be a clarification on the purpose of the active participation, emphasizing that the students should regard the format as supportive for their future skills rather than intimidating.

In the resources items, positive responses increased substantially, with 82% of respondents providing positive ratings and nearly half selecting Strongly Agree (Figure 5). This concentration of positive responses indicates that access to structured resources is perceived as highly beneficial. However, the largest proportion of neutral and negative responses within this group concerned the availability of physical guidance during the poster preparation phase (Figure 3). A likely contributing factor is that student groups were encouraged to work independently, combined with the practical limitation that only a single teaching staff member was available during the session. This may have reduced students’ perceived accessibility to immediate, hands-on support when uncertainties arose. In the future, increasing the availability of instructional staff, such as assigning two teachers rather than one, could help mitigate this issue, particularly during phases requiring practical assistance or conceptual clarification.

The merged approach yields a combined positive response rate of 74%. This indicates high effectiveness and a more consistent positive perception among participants (Figure 5). Despite the generally strong endorsement of the merged approach, one item, “I learned more histology this way”, showed a somewhat higher proportion of negative responses (20%) compared with the other statements (Figure 4). It is noteworthy that the majority of students still provided positive ratings (69% agreement or strong agreement), and learning gain is inherently difficult for students to self-assess accurately immediately after an activity [21]. The somewhat elevated negative responses may therefore reflect uncertainty rather than a substantive limitation of the merged approach. Importantly, all other items relating to activation, interest, enjoyment, and clarity during discussion received consistently high endorsement, indicating strong perceived pedagogical value overall. Hence, in the future, examination results should be documented and compared to earlier exams to offer objective evidence clarifying whether the merged approach not only enhances engagement but also translates into measurable improvements in histology learning.

All in all, the merged approach’s key positive response from the students, as presented in the table below (Table 1), was able to foster active engagement, provided a sense of contribution, and yielded high attendance with a sense of high accessibility of the digital resources. This aligns with theoretical models emphasizing that effective learning environments integrate metacognitive engagement with appropriate instructional support [22]. Importantly, participants strongly agreed that clinically relevant aspects were clearly emphasized, reinforcing the educational value of the discussion-driven format. Bridging the basic sciences with practical applicability in dental education has been emphasized in recent studies [23].

The present findings align with previous research in dental and medical education showing that blended learning, flipped classrooms, and team-based approaches are associated with increased student engagement and satisfaction compared with traditional lecture-based formats [24,25,26]. These studies emphasize that combining digital preparatory materials with interactive, application-oriented classroom activities promotes deeper learning and perceived relevance, which is consistent with our results.

Furthermore, the strong emphasis students placed on digital accessibility and structured guidance reflects national trends in student expectations. Data from the Norwegian national student survey (Studiebarometeret) highlight the importance of organized teaching, accessible resources, and academic support for overall satisfaction [27]. Our findings correspond with these priorities, while also identifying supervision during active group work as an area for potential improvement.

The questionnaire was developed by the last author and discussed with the senior co-authors to ensure relevance. It was internally reviewed and tested by the student co-authors for clarity; however, it was not formally pilot-tested or psychometrically validated, which constitutes a study limitation. Furthermore, this type of study has its reliance on self-reported perceptions, which are inherently subjective and may not accurately reflect actual learning outcomes. Students’ evaluations can be influenced by factors unrelated to instructional quality, such as workload, timing within the semester, personal stress levels, or preferences for teachers and teaching approaches [28]. Logistical constraints also introduced variability, as on several occasions, two groups were merged to fit the scheduled timetable, and the resulting change in group dynamics led to more vocal participation from certain students while potentially inhibiting shyer individuals. Such variation may have influenced the perceived quality of discussion and the overall learning experience. Also, this study lacked objective performance measures such as examination data, limiting the ability to determine whether perceived benefits translated into measurable learning gains. The questionnaire also did not include an open-ended response option, preventing participants from providing extra qualitative feedback that could have clarified or contextualized their ratings. Finally, this study included the entire cohort of master-level dental students enrolled in the course (n = 51), with 45 respondents (88% response rate). Although the absolute number is modest, as this was the first implementation of the merged teaching approach, it represents the full population available within a single academic semester and reflects a high participation rate. However, use of a single cohort and modest sample size restricts generalizability, indicating that the findings should be interpreted cautiously and supplemented with further evidence in future research.

## 5. Conclusions

The findings demonstrate a clear and statistically significant overall endorsement of the merged teaching approach in dental histology, in which traditional didactic components and modern active-learning strategies were structurally and chronologically integrated. Students reported high levels of engagement, perceived learning enhancement, and recognition of clinical relevance.

The results indicate that combining structured digital preparatory materials with interactive, discussion-driven sessions and collaborative poster-based activities promotes deeper cognitive processing and reflective engagement. This blended format strengthens students’ ability to integrate molecular and developmental principles with tissue architecture and clinical implications. By emphasizing developmental chronology, microstructural organization, and applied clinical examples, the approach bridges foundational biological sciences with practical odontological competence, thereby supporting diagnostic reasoning and clinical decision-making.

Based on these findings, educators are encouraged to organize histology teaching chronologically, integrate preparatory digital resources with active in-class application, maintain a continuous focus on clinical relevance, and provide structured guidance during collaborative learning activities. Although further research incorporating objective performance measures and larger cohorts is warranted, the present results suggest that a blended and chronologically structured teaching model may enhance educational outcomes and contribute to improved clinical preparedness in dental training.

## Figures and Tables

**Table 1:** Strongest findings from the student perception questionnaire (*n* = 45).

Item	Strongly Agree (%)	Agree (%)	Interpretation
Attended almost all sessions	93	7	Very strong attendance
Contribution to group	42	44	High collaborative confidence
Online lectures easily available	62	27	Very high digital accessibility
Felt activated and engaged	31	47	Strong engagement effect
Clinical relevance emphasized	31	47	Clear clinical integration

## Data Availability

The original contributions presented in this study are included in the article. Further inquiries can be directed to the corresponding author.
